# Poor Outcome of Metastatic Cutaneous Squamous Cell Carcinoma: A Swedish Retrospective Study

**DOI:** 10.2340/actadv.v106.44057

**Published:** 2026-02-23

**Authors:** Charlotta L. PALMQVIST, Mathias VON BECKERATH

**Affiliations:** 1 Visby Regional Hospital, Visby; 2 Karolinska institute, Stockholm, Sweden

**Keywords:** metastasis, cutaneous squamous cell carcinoma, skin, SCC, metastatic

## Abstract

The incidence of cutaneous squamous cell carcinoma is increasing rapidly, and 1.5–5% of cases develop metastatic disease, which is associated with a markedly worse prognosis. This study aimed to describe the characteristics of the primary tumour, the patients, and the metastases, as well as treatment and outcomes in patients with metastatic cutaneous squamous cell carcinoma. A total of 137 patients with a diagnosis of metastatic cutaneous squamous cell carcinoma within the Stockholm region during 2012–2020 were identified and included. Median age was 81; 79% were male; 70% had a WHO performance status of 0–1. 50% of the primary tumours were classified as high-risk tumours according to the Brigham and Women’s Hospital system. The median time between diagnosis of the primary tumour and the diagnosis of regional metastases was 7 months and 90% of the metastases were diagnosed within 2 years. Most patients were treated with a combination of surgery and radiotherapy. Overall survival at 2 years was 56%. This study shows that metastatic cutaneous squamous cell carcinoma occurs in elderly but otherwise generally healthy patients, that primary tumours with a high risk of causing metastases seem hard to identify, and that metastases occur within 2 years after primary tumour diagnosis. The patient group overall has a high mortality rate, particularly among those who do not undergo surgery.

## SIGNIFICANCE

Cutaneous squamous cell carcinoma is the second most common cancer in Sweden, its incidence is increasing, and metastasized disease has a high mortality rate. This is the first published study on metastatic cutaneous squamous cell carcinoma in Sweden and reports descriptions of this disease.

Cutaneous squamous cell carcinoma (cSCC) is the second most common cancer in Sweden and its incidence is increasing rapidly. In Sweden during 2023, over 12,000 new cases of cSCC were reported and its incidence is calculated at 137 new cases per 100,000 people for men and 107 for women (1). Cumulative UV radiation, old age, and male sex are the most important risk factors for developing cSCC (2–8). The increasing incidence is believed to be caused by an ageing population and increased exposure to UV radiation. Approximately 80% of cSCC cases are localized to the head and neck region, accounting for a large proportion of consultations in the ENT department (5, 8–10).

For the majority of patients, the disease is easily treated by surgical excision. Unfortunately, 1.5–5% of cSCC metastasize and in these cases the patients’ prognosis worsens radically (11–22).

Risk factors for developing metastatic cSCC (mcSCC) include tumour size ≥ 2 cm, invasion depth, degree of differentiation, perineural invasion, intravascular invasion, subcutaneous invasion, immunosuppression, tumour relapse, and tumour location (11, 14, 15, 19, 23–30). In the national guidelines of Sweden, the Brigham and Women’s Hospital (BWH) staging system is used to identify high-risk tumours postoperatively (16, 31). For mcSCC the TNM-system (UICC 8) is used.

The great majority of metastases occur within the first 2 years after the diagnosis of the primary tumour and most often affect the parotid gland or the lymph nodes of the neck (14, 15, 18–20, 26, 32, 33). A literature review from 2018 stated that disease-specific 5-year survival was between 58% and 83% and a large Australian retrospective cohort from 2024 showed a 5-year Disease Specific Survival (DSS) of 77.6% (22, 34). There is currently no reported survival rate in Sweden, but unpublished Swedish data point towards a 52% overall survival within 18 months, which is similar to the results of a recent Finnish study by Knuuttila et al. (18). Little is known about mcSCC in Sweden as it is not recorded in a national registry as a unique diagnosis and no recent studies have been published.

As we expect an even higher incidence of cSCC in the future we can assume that mcSCC will cause a significant patient burden in ENT cancer departments. In this study we aim to describe the patient characteristics, tumour characteristics, treatment, and outcome of Swedish patients with mcSCC in Stockholm region during the study period 2012–2020.

## MATERIALS AND METHODS

The study was approved by the Swedish ethical committee. Stockholm region’s charting system *Take Care* was searched for adult patients who were diagnosed with metastasized or locally advanced cSCC between 2012 and 2020. The charting system was searched for the ICD-10 diagnostic code C44 (skin tumour) in combination with C77 (lymph node metastases) and C44 in combination with surgical codes ELB40 (parotic resection), ELB50 (parotidectomy), or PJD51 (neck dissection). Patients were included if they had cSCC in the head and neck area and locally advanced or metastatic disease. Their medical records were reviewed, and data (see list of variables in **Table I**) were collected.

**Table I:** Patient characteristics

Variable	*n* (%)
Number of patients	137 (100)
Age, years	
≤ 60	4 (3)
60–80	61 (45)
> 80	72 (53)
Sex	
Female	29 (21)
Male	108 (79)
Smoking	
Active smoker	8 (6)
Previous smoker	58 (42)
Never smoked	71 (52)
Immunosuppression	
No	103 (75)
Yes (total)	34 (25)
CLL	18
Organ transplant	8
Other blood disease	6
Other	2
WHO performance status	
Ps 0	66 (48)
Ps 1	30 (22)
Ps 2	24 (18)
Ps 3	15 (11)
Ps 4	2 (1)
Previous diagnosis of cSCC	
Yes	44 (32)
No	93 (68)

## RESULTS

The search identified 309 patients of whom 172 were excluded because they had cancer diagnoses other than cSCC, their primary tumours were located outside the head and neck area, or they were treated for their metastasized or locally advanced cSCC at a hospital outside of the study period or outside of the Stockholm region. This resulted in 137 patients who were included in the study.

### Patient characteristics

The average age at the time of the primary tumour diagnosis was 80 years (81 median). On average each patient had 3.4 comorbidities; 70% had a WHO performance status of 0–1 and 30% 2–4. The remaining patient characteristics are summarized in Table I.

### Primary tumour characteristics

In 2/137 cases the primary tumour was unknown, resulting in 135 primary tumours to analyse. According to the BWH system 50% were classified as high-risk tumours after the primary surgery. The remaining tumour characteristics are summarized in **Table II**.

**Table II:** Primary tumour characteristics

Variable	*n* (%)
Number of known primary tumours	135 (100)
Site	
Ear	28 (21)
Temple	26 (19)
Cheek	21 (16)
Scalp	18 (13)
Periauricular	14 (10)
Forehead	9 (7)
Lip (vermilion)	7 (5)
Neck	6 (4)
Nose	5 (4)
Chin	1 (1)
Size	
< 2 cm	51 (39)^a^
≥ 2 cm	80 (61)^a^
unknown	4 (3)
Degree of pathological differentiation	
Low	52 (41)^a^
Moderate	66 (52)^a^
High	9 (7)^a^
Unknown	8 (6)
Perineural invasion	
Yes	21 (16)
No/unknown	114 (84)
Growth beyond fat	
Yes	51 (38)
No/unknown	84 (62)
BWH^b^	
T1	27 (21)^a^
T2a	38 (30)^a^
T2b	57 (45)^a^
T3	5 (4)^a^
Unknown	3
Radical margins	
Positive	49 (39)^a^
Negative	76 (61)^a^
Unknown	2 (1)
Non-surgical treatment of primary tumour	8 (6)

a Percentage excludes the unknown data.

b 7 lip tumours excluded.

### Metastases characteristics

The average time between diagnosis of the primary tumour and the diagnosis of regional metastases was 11 months and median time 7 months; 90% of the metastases were diagnosed within 2 years. The average time between the diagnosis of the primary tumour and the diagnosis of distant metastases was 26 months and median time 16 months. The average and median age at the diagnosis of regional metastases was 82 years. The average and median age at the time of distant metastases was 81 and 82 years respectively. The average and median disease-free survival (DFS) was 11 and 6 months respectively. The remaining characteristics of the metastases are summarized in **Table III**.

**Table III:** Metastases characteristics

Variables	*n* (%)
Type of advanced cSCC	
Metastases	128 (93)
Regional metastases	123
Distant metastases only	5
Distant metastases	29
Locally advanced disease without metastases	9^a^ (7)
cTNM^b^	
T1	53 (39)
T2	19 (14)
T3	60 (44)
T4	1 (1)
Tx	4 (3)
N0	16 (12)
N1	21 (15)
N2a	4 (3)
N2b	23 (17)
N2c	5 (4)
N3a	0 (0)
N3b	68 (50)
M0	119 (87)
M1	14 (10)
Mx	4 (3)
Stage (excluding lip)	
I	0 (0)
IIa	12 (9)
IIb	0 (0)
IIIa	16 (12)
IIIb	27 (21)
IV	75 (58)
Stage lip cancer	
I	0
II	0
III	3 (43)
IVa	2 (29)
IVb	2 (29)

a Locally advanced disease was defined as at least T3 tumours that needed extensive surgery in the ENT department or radiotherapy in the oncology department.

b Classified at the multidisciplinary cancer conference (MCC) after the metastases surgery, or at the primary MCC if the patient did not undergo surgery, or at the MCC after the major surgery if locally advanced tumour without metastases.

### Treatment

**Fig. 1:**
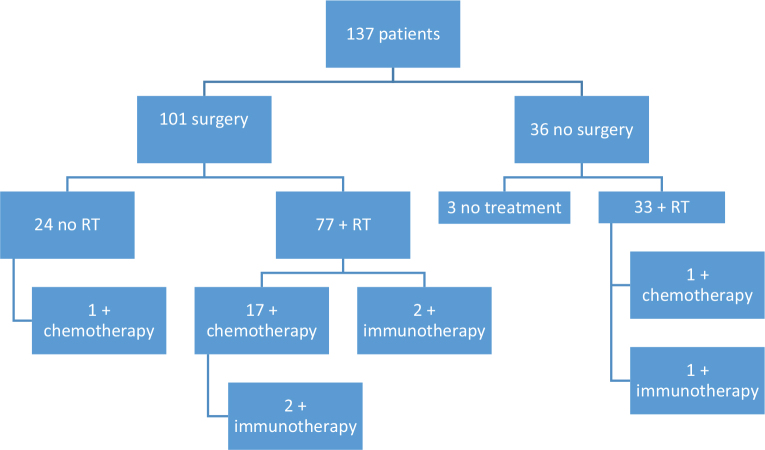
Treatment.

A total of 101 patients received surgery due to regional metastases or locally advanced disease, 76% (94/123) of those with regional metastases and 78% (7/9) of those with locally advanced tumours. Radical excisions were performed in 45% (45/101) of the surgeries. 80% (110/137) of the patients had radiotherapy (RT); 14% (19/137) had chemotherapy; 4% (5/137) had immunotherapy. The patients’ treatments are shown in **Fig. 1**. The rest of the treatment data are summarized in **Table IV**.

**Table IV:** Treatment

Variable	*n* (%)
Type of treatment	
Surgery only	23 (17)
Surgery + RT	58 (42)
Surgery + RT + chemotherapy	15 (11)
Surgery + RT + chemotherapy + immunotherapy	2 (1)
Surgery + RT + immunotherapy	2 (1)
Surgery + chemotherapy	1 (1)
RT only	31 (23)
RT + chemotherapy	1 (1)
RT + immunotherapy	1 (1)
None	3 (2)
Number of patients who underwent surgery	101 (74)
Type of surgery	
Parotidectomy + neck dissection^a^	48 (48)
Parotidectomy + lumpectomy^a^	13 (13)
Neck dissection only	21 (21)
Parotidectomy only	7 (7)
Other surgery	12 (12)
Location site of the metastasis^b^	
Parotic gland	49 (49)
Region I	20 (20)
Region II	32 (32)
Region III	12 (12)
Region IV	3 (3)
Region V	15 (15)

a Neck dissection is defined as removal of 3 or more regions. Lumpectomy is defined as removal of 2 or fewer regions. Percentage is calculated from the patients who underwent surgery.

b From the pathological records of the patients who underwent surgery (total 101 patients). The same patient can appear in multiple categories.

### Outcome

At the end of the study period (July 2023) an 80% (109/137) mortality rate was seen in the study population. Among the immunosuppressed an 88% (30/34) mortality rate was seen. Average time between the primary diagnosis of cSCC and death was 33 months and median 22 months. Average time between the identification of regional metastases and death was 21.5 months and median 13 months. Average time between diagnosis of distant metastases and death was 14 months and median 5 months; 51% (56/109) of the deaths were determined to be directly related to the cSCC diagnosis. In 18 cases the cause of death was unknown. Excluding unknown causes of deaths, 62% (56/91) of the deaths were assessed to be caused by cSCC. Other outcomes are summarized in **Table V**.

**Table V:** Outcome

	*n* (%)
Overall survival 2 years	
After primary tumour	76/135^a^ (56)
After regional metastases	51/123 (41)
After distant metastases	7/29 (24)
Overall survival 2 years after primary diagnosis	
Surgery, no RT	12/23^b^ (52)
Surgery + RT	54/77 (70)
RT, no surgery	10/32^c^ (31)
No treatment	0/3 (0)
Overall survival 2.5 years	
After primary tumour	68 /135^a^ (50)
After regional metastases	45/123 (37)
After distant metastases	5/29 (17)
Overall survival 2.5 years among immunosuppressed	
After primary tumour	14/34 (41)
After regional metastases	9/30 (30)
After distant metastases	2/8 (25)
Overall survival 2.5 years after primary diagnosis	
Surgery, no RT	11/23^b^(48)
Surgery + RT	49/77 (64)
RT, no surgery	9/32^c^ (28)
No treatment	0/3 (0)
Dead in July 2023	109/137 (80)
Dead in July 2023 among immunosuppressed	30/34 (88)

a 2 patients excluded due to unknown data concerning primary diagnosis.

b 1 excluded due to unknown primary.

c 1 excluded due to unknown primary.

## DISCUSSION

Cutaneous SCC is the second most common cancer in Sweden, its incidence is increasing, and metastasized disease has a high mortality rate. This is the first published study on mcSCC in Sweden and reports descriptions of this disease.

The most common sites of primary tumours among mcSCC in Sweden were ear and temple. This is similar to studies from other countries (11, 18).

The median age of the patient group was 81 but 70% were assessed as WHO-performance status 0-1, indicating that these patients are elderly but relatively healthy at the time prior of the treatment. Despite this, overall survival after 2.5 years from diagnosis of regional metastases was only 37%, which suggests the aggressiveness of the disease and/or the lack of effective treatment. With immunosuppression the prognosis is even worse.

In Sweden the BWH system is used to identify high-risk primary tumours. Other studies have reported a low sensitivity but a high specificity of the BWH system (16, 35–37). Knuuttila et al. could not find a correlation between BWH and prognosis (18). In our study only 50% of the primary tumours were assessed as BWH high-risk, which supports these other studies’ results reporting its low sensitivity. We encourage the development of a more sensitive risk stratification system to find high-risk primary cSCC.

Median time between primary tumour and regional metastases was 7 months and 90% of the metastases were diagnosed within 2 years. This data is in line with earlier studies, including Knuuttila et al., which reported 6.6 months and 85% respectively (13, 14, 18, 21, 26, 32, 38). This suggests that close monitoring of patients during the first 2 years following a high-risk cSCC is essential for the early detection of metastases.

Most metastases were located in the parotic gland and region I–II, which concurs with earlier studies (13). 58% of our patients were classified as TNM stage IV after their surgery. The majority (87%) were due to clinically extracapsular growth of the metastases. Ebrahimi et al. found that 81% of their study population had metastases with extracapsular growth and that this significantly affected the patient outcome. However, they suggested that the presence of extracapsular growth upstaged too many patients to TNM stage IV, despite many having curable disease (34).

The patients received different combinations of treatment, mostly surgery with adjuvant radiotherapy. Chemotherapy and immunotherapy were never given as single therapy, but were given as an addition to surgery and/or radiotherapy. The highest overall survival was among the patients receiving surgery + radiotherapy, where 64% were alive 2.5 years after the primary diagnosis. The overall survival was 48% for patients who received only surgery and 28% for those who received only radiotherapy. Obviously, the patient’s comorbidities and level of functioning are taken into consideration when choosing which treatment a patient will receive, but our results suggest a higher mortality rate without surgery. Earlier studies have also shown better prognosis if patients receive both surgery and radiotherapy compared with surgery alone (21, 34, 39).

Our overall survival rate (OS) at 2 years was 56% from primary tumour diagnosis and 41% from metastases diagnosis. These numbers are similar to the results of Knuuttila et al. where 2-year OS from primary tumour diagnosis and metastases diagnosis was 63.8% and 43.4% respectively (18). Other studies have shown OS at 2 years of 50–66% from time of metastases (32, 38).

Study limitations are mostly related to those expected in a retrospective cohort study. Some data were missing, and patients may have not been identified due to incorrect ICD coding of patients during treatment. Some patients may never have been referred to a hospital for treatment but rather received palliative treatment at home. Cause of death was rarely confirmed by autopsy and was therefore difficult to relate to the cSCC diagnosis. Another limitation is that only 4% of our patients received immunotherapy as this was not part of the European guidelines during the study period. Given that 32% of the patients had a medical history of a previous cSCC, the tumour defined as the primary source of metastasis may have been incorrectly identified.

### Conclusion

Our study, which is the first reported in the Swedish population, shows that mcSCC occurs in elderly but otherwise generally healthy patients, that primary tumours with a high risk of causing mcSCC seem hard to identify, and that metastases occur within 2 years after primary tumour diagnosis. The patient group overall has a high mortality rate, particularly among those who do not undergo surgery. We encourage the development of a more sensitive risk stratification system to find high-risk primary cSCC, and close monitoring of patients during the first 2 years following a high-risk primary cSCC.
